# Availability of arsenic in human milk in women and its correlation with arsenic in urine of breastfed children living in arsenic contaminated areas in Bangladesh

**DOI:** 10.1186/1476-069X-13-101

**Published:** 2014-12-04

**Authors:** Md Rafiqul Islam, John Attia, Mohammad Alauddin, Mark McEvoy, Patrick McElduff, Christine Slater, Md Monirul Islam, Ayesha Akhter, Catherine d’Este, Roseanne Peel, Shahnaz Akter, Wayne Smith, Stephen Begg, Abul Hasnat Milton

**Affiliations:** Centre for Clinical Epidemiology & Biostatistics (CCEB), The School of Medicine and Public Health, Faculty of Health, The University of Newcastle, Lot 1 Kookaburra Circuit, New Lambton Heights, NSW 2305 Australia; Wagner College, 631 Howard Avenue State, Island, NY 10301 USA; Division of Human Health, International Atomic Energy Agency, Vienna, Austria; Centre for Nutrition and Food Security, International Centre for Diarrhoeal Diseases Research, Bangladesh, Mohakhali, Dhaka, 1212 Bangladesh; Department of Obstetrics and Gynaecology, John Hunter Hospital, Hunter New England Area Health Services, 2 Lookout Road, New Lambton Heights, NSW 2305 Australia; Department of Paediatrics, Institute of Child and Mother Health, Matuail, Dhaka, Bangladesh; Department of Environmental Health, NSW Health, Sydney, NSW Australia; School of Rural Health, LaTrobe University, Bendigo, VIC Australia

**Keywords:** Arsenic, Human milk, Bangladesh

## Abstract

**Background:**

Early life exposure to inorganic arsenic may be related to adverse health effects in later life. However, there are few data on postnatal arsenic exposure via human milk. In this study, we aimed to determine arsenic levels in human milk and the correlation between arsenic in human milk and arsenic in mothers and infants urine.

**Methods:**

Between March 2011 and March 2012, this prospective study identified a total of 120 new mother-baby pairs from Kashiani (subdistrict), Bangladesh. Of these, 30 mothers were randomly selected for human milk samples at 1, 6 and 9 months post-natally; the same mother baby pairs were selected for urine sampling at 1 and 6 months. Twelve urine samples from these 30 mother baby pairs were randomly selected for arsenic speciation.

**Results:**

Arsenic concentration in human milk was low and non-normally distributed. The median arsenic concentration in human milk at all three time points remained at 0.5 μg/L. In the mixed model estimates, arsenic concentration in human milk was non-significantly reduced by -0.035 μg/L (95% CI: -0.09 to 0.02) between 1 and 6 months and between 6 and 9 months. With the progression of time, arsenic concentration in infant’s urine increased non-significantly by 0.13 μg/L (95% CI: -1.27 to 1.53). Arsenic in human milk at 1 and 6 months was not correlated with arsenic in the infant’s urine at the same time points (r = -0.13 at 1 month and r = -0.09 at 6 month). Arsenite (AsIII), arsenate (AsV), monomethyl arsonic acid (MMA), dimethyl arsinic acid (DMA) and arsenobetaine (AsB) were the constituents of total urinary arsenic; DMA was the predominant arsenic metabolite in infant urine.

**Conclusions:**

We observed a low arsenic concentration in human milk. The concentration was lower than the World Health Organization’s maximum permissible limit (WHO Permissible Limit 15 μg/kg-bw/week). Our findings support the safety of breastfeeding even in arsenic contaminated areas.

## Background

Chronic arsenic exposure via drinking water has been considered a serious public health problem in many countries [1]. More than 100 million people in the world are chronically exposed to arsenic and nearly one quarter to one half of this total are living in Bangladesh [2]. Exposure to this known toxicant and carcinogen brings about profound adverse health outcomes in humans [2–5]. Evidence exists for associations between maternal arsenic exposure and foetal loss, impaired foetal growth, reduced thymic function in infants, and increased infant morbidity and mortality [6, 7]. Infants that are solely breastfed showed potential for exposure to heavy metals including arsenic via breast milk [8] which might increase the risk of adverse health consequences.

Early life and in-utero arsenic exposure in children and foetuses is a growing concern in terms of development of chronic diseases in later life [9, 10] and has drawn increasing attention [11]. Exposure effects including windows of exposure in young and school aged children have not been investigated adequately [1, 12] and it remains inconclusive whether arsenic is an immediate or long term health threat to children, infants, babies and foetuses [13]. It is generally known that exposure to environmental contaminants during key developmental stages can cause toxic injury to brain tissue [14]. There is substantial evidence for having similar concentrations of arsenic in cord and maternal blood and arsenic can readily cross the placenta during the late gestational period [15]; this may cause brain damage in the developing foetus. This prenatal exposure is found to be associated with adverse effects in early childhood and both pre and postnatal exposure can contribute to disease in later life [16]. There is inconclusive data on the presence and amount of postnatal exposure to arsenic via breast milk [7, 17] and less information on its metabolism in young people. Efficient arsenic methylation to dimethyl arsinic acid (DMA) increases arsenic excretion from the body which in turn reduces the health risks [18], while higher monomethylarsonic acid (MMA) content in urine is associated with increased risk of adverse health outcomes in adults [19]. It is thus essential to know what amount of arsenic passes through breast milk to breastfeeding children living in arsenic contaminated areas. This is particularly important for assessing the World Health Organization’s (WHO) policy for exclusive breastfeeding in infants until 6 months and breastfeeding predominantly until 9 months [20]. We therefore aimed to detect the amount of arsenic in human milk of lactating mothers and relate this to corresponding maternal and children’s urinary arsenic in arsenic contaminated areas in Bangladesh; we also aimed to determine the arsenic species in these samples.

## Methods

This longitudinal study was conducted over a period of 13 months between March 2011 and March 2012 in Kashiani Upazila (Sub-district), Gopalgonj, Dhaka, Bangladesh. The whole Gopalgonj district is arsenic contaminated; 94% of ground water samples that were surveyed previously contained arsenic over the permissible limit of 50 μg/L [21] and the study subdistrict is a known contaminated area. The subdistrict is situated about 200 kilometers south west of the country’s capital city, Dhaka. The subdistrict is comprised of 45,170 households with a total population of 228,647 over 299.64 square kilometres and is divided into 14 unions. A union is the smallest administrative geographical unit of the country. On the basis of convenience and accessibility, we purposively selected 3 unions initially to recruit eligible participants.

A total of 120 mother-infant pairs were recruited prospectively. Any new, healthy, mother and infant pair who had lived in the study area for a period of 12 months and could provide the required information, body measurements and biological samples, was eligible. Of the 120 prospectively selected mother-baby pairs, 30 were randomly selected and approached to provide human milk samples at 1, 6 and 9 months postnatal as well as maternal and infant urine samples at 1 and 6 months. Also, from these 30 mother-baby pairs, 12 were randomly selected for urinary arsenic speciation.

### Ethics

Ethical approval was obtained from the Human Research Ethics Committee (HREC), The University of Newcastle, Australia (number H-2010-1211). The study also received ethical approval from Bangladesh Medical Research Council (BMRC) (number BMRC/HNPSP/2010-2013/372). Written informed consent was obtained from all participating mothers or from the head of the household.

### Data collection procedure

Three trained female Field Research Assistants screened households in the selected unions using a list of pregnant mothers who would give birth in the following three month period and who gave birth in the previous one month period from the day of household visit. The list of potential pregnant mothers and mother-newborn pairs was supplied by the FWAs (FWA: Family Welfare Assistant) and supervisors (FPI: Family Planning Inspector, AFPI: Assistant Family Planning Inspector) of the Ministry of Health and Family Welfare, Government of Bangladesh. Since the literacy rate is low in the selected sampling areas in Bangladesh, the study information sheet was read out to potential participants, and the objectives and expectations of the study were comprehensively explained. At each contact point with the household, the Field Research Assistants reminded the household members of the government policy not to drink or use water from tubewells marked with a red colour as these contain arsenic at or above the WHO (World Health Organization) safety limit for drinking water for Bangladesh (50 μg/L). The study information sheet was then left with the potential participants, and after 48 hours they were contacted either via mobile phone or by household visit; trained field workers then obtained written informed consent from the participating mother or her husband/guardian. Subsequently, Field Research Assistants collected questionnaire information, obtained biological samples and performed body measurements of the mother-baby pairs.

### Exposure measurement

A single tubewell water measurement and current household drinking water samples drawn from that well were used to measure current arsenic exposure for each participating mother-baby pair following standard procedures [22]. Water arsenic analyses were performed using continuous flow hydride generation atomic fluorescence spectroscopy (HGAFS). The minimum detection level of arsenic by this method is 1 μg/L.

### Sample collection and preservation

#### Drinking water

Drinking water samples were collected once only in a 50 ml plastic bottle from each survey household at the time of initial contact and participant recruitment. The plastic bottles were pre-acid washed in the NGO forum for Drinking Water (a leading national Non-Government Organization) laboratory, Dhaka, Bangladesh. The field workers pumped out about a litre of water from the tubewell before collecting water samples in the bottle. The bottle was then sealed tightly with parafilm tape and kept in an ice box in the field after labelling.

#### Human milk

Approximately 10 ml of human milk sample was collected from each participating mother at each time point in prewashed and dried 20 ml plastic containers. The breast milk samples were collected by the interviewing field research assistants in a separate closed and or locked room under adequate privacy. The sampling time points (1, 6 and 9 months) are transition points for neonate to infant, exclusive breastfeeding to complementary feeding, and continuing breastfeeding with complementary feeding, respectively. After collection, the containers were sealed with parafilm tape and preserved in an ice cold box in the field after labelling.

#### Urine

Mothers’ and children’s day time urine samples were collected in prewashed and dried 20 ml plastic containers when the child was 1 and 6 months old. A PUC (paediatric urine collection) bag was attached around the child’s external genitalia and the Research Assistants encouraged the mother to breastfeed her infant. The Research Assistants waited in the survey household until the child urinated in the PUC bag and transferred the samples from PUC bag to the 20 ml plastic container; these were stored in an ice box in the field following labelling. Upon receipt at the field office, all samples (water, milk, urine) were stored in a freezer at -20°C until they were transferred to the reference laboratory in the USA for arsenic analysis.

#### Sample analysis

All the biological samples and drinking water samples were analysed in the Department of Chemistry Laboratory, Wagner College, 631 Howard Avenue state island, NY 10301, United States and urinary arsenic speciation was adjusted by specific gravity.

### Arsenic analysis in human milk

#### Sample digestion

Human milk samples were digested in *Ultrex* high purity (99.99% pure) nitric acid HNO_3_ (J.T. Baker Chemical Co., USA). An aliquot of 1–2 ml sample was taken in a polyethylene digestion tube and 3mls of HNO_3_ were added to it. In addition, 2 mls of high purity (99.99% pure) H_2_O_2_ were added to the sample. The mixture was heated in a microwave oven (MARS X, CEM Corporation., USA) at 180°C at 1600 watt power for 20 minutes, and cooled for 10 minutes; this cycle was repeated 2 more times. The solution was reduced to approximately 2 mls after heating. The final volume of the solution was adjusted to 5 ml with ultra-purified (18.2 MΩ) water. For each batch of sample digestion, a blank was prepared in exactly the same manner with all reagents in the tube except a sample.

#### Analytical technique of breast milk

Arsenic (As) analysis in human milk samples was carried out by graphite furnace atomic absorption spectroscopy (GF-AAS) (Perkin Elmer, Model Analyst 800) with Zeeman background correction and high energy electrode-less discharge lamps. The matrix modifier for As analysis consisted of a solution containing 1% (w/v) Ni(NO_3_)_2▪_6H_2_O and 0.1% Triton X-100. High purity (99.99%) Ni(NO_3_)_2_.6H_2_O, 2% (w/v) (Sigma-Aldrich Co., USA) in 18 MΩcm^-1^ water was used throughout the analysis.

Calibration standards were prepared fresh daily using a 1000 mg/l standard As solution (Perkin-Elmer Co., USA) and 0.1% Triton X-100 as diluent. For As analysis the matrix modifier consists of 1% Ni(NO_3_)_2_.6H_2_O (Sigma-Aldrich Co., USA), 0.1% Triton X-100 (Fisher Scientific Co., USA). For As analysis, sample and matrix modifiers are co-injected in the graphite furnace.

All samples, method blanks, quality control (QC) standards were loaded in an auto sampler tray (AS 60) (Perkin-Elmer Co., USA) in the GF-AAS system. The minimum detection level with this method was 0.50 μg/l for both urine and breast milk.

#### Arsenic analysis in urine samples of mothers and infants

The urine samples were diluted with distilled deionized water in 1:4 or 1:5 ratio to bring the concentration of arsenic within the range of the arsenic calibration curve. Standard solutions were prepared by diluting 1000 mg/l stock solution (Perkin-Elmer Co., USA) with control urine samples collected from laboratory personnel not exposed to arsenic from drinking water. The matrix modifier for arsenic analysis in urine sample was a solution consisting of 1% K_2_S_2_O_8_ and 1% Pd(NO_3_)_2_ solution prepared in 0.10% HNO_3_. For method validation control reference samples of SRM 2670 (NIST) and *Seronorm* (Sero, Norway) urine samples were analyzed under identical analysis conditions.

#### Quality control

The spectrophotometers AAS and AFS were calibrated everyday with standards prepared from stock 1000 ppm As solution from Perkin-Elmer Co. (USA). The software alerts the analyst to any concentration over the calibration linear range and the sample is subsequently diluted. The AFS was calibrated in ppb (microgram of arsenic per liter of water) unit and the concentration of arsenic in all samples was directly read from the spectrometer. For quality control (QC), the QC standards and reagent blanks were analyzed at regular intervals. All standards and the reagents were prepared fresh daily. Standard reference materials (SRM 1643d, SRM 1640, SRM 2670) from the National Institute of Standards and Technology (NIST, USA) were used as controls for precision and accuracy checks of data and validation of analytical methods.

#### Other information

Research Assistants collected information on socio-demographics, drinking, bathing, washing, cooking, and history of water use by face to face interview using a structured questionnaire. Information on postnatal food intake including use of colostrum, external and or complementary foods and infectious disease morbidity at 3, 6 and 9 months was also collected.

#### Statistical analysis

Data were analysed using STATA version 11 supplied by STATA Corporation, TX, USA. Frequency tables were obtained to check missing data, out of range values and to assess distributions of continuous variables. Arsenic concentration in drinking water that was collected at initial contact with the household was categorised as <50 μg/L and ≥50 μg/L since arsenic at 50 μg/L of drinking water is the maximum permissible limit for Bangladesh, set by the World Health Organization [21].

Summary statistics including mean, median and ranges for the arsenic in human milk and in infant’s urine were presented. Scatter plots were drawn for arsenic concentration in human milk and urine samples at all the time points collected. We also performed simple linear regression analysis. To observe any significant increment or decrement of arsenic concentration in human milk over time using mixed model estimates in linear regression.

## Results

From the 30 randomly selected mother-infant pairs, human milk samples from 29 mothers at 1 month (23 households with arsenic at <50 μg/L), 25 mothers at 6 months (19 households with arsenic at <50 μg/L) and 19 mothers at 9 months (14 households with arsenic at <50 μg/L) were available for arsenic analysis. Urine samples from 29 mothers and 29 babies at 1 month, and 27 mothers and 27 babies at 6 months were available for arsenic analysis. Of these urine samples from both the mother-baby pairs, all twelve randomly selected samples were available for arsenic speciation at 1 and 6 months.

During the initial stage of the study, six (21%) of the households’ drinking water contained arsenic at ≥50 μg/L. The study mothers were relatively young with a mean age of 24.6 years at the time of initial biological sample (human milk, urine) collection i.e. at 1 month of child’s age. About 62% (18) of newborns were offered external food or drink such as normal water, sugar, glucose or crystalline sugar water, honey, powdered and cow’s milk in the three days after birth. At 6 months of infant’s age, 10% (3) of the infants were not on any breastfeeding and 48% (14) had started feeding with formula milk using spoon. Almost all the children (97%) were given water and about 41% (12) were offered fruit juice to drink at 6 months of age. Semolina, sweet foods and eggs were predominantly (62%, 38% and 34%, respectively) given to the infants at 6 months of age. Table 1 depicts the characteristics of the participating mother-infant pairs.

**Table 1:** **Demographic and other characteristics of participating mother baby-pairs (N = 29)**

Mother			Infant		
Characteristics	Values	Range	Characteristics	Values	Range
Age in years at initial survey:	24.6 ± 5.0	18-40	-	-	-
-	-	-	Child’s gender, n (%):		
			Male:	15 (51.7)	-
			Female	14 (48.3)	-
-	-	-	Child’s birth wt. in kg :	3.2 ± 0.6	2-4
			Missing, n (%):	19 (65.5)	-
Religion, n (%):			^§^Given any drink		
Muslim:	19 (65.5)	-	within 72 h of birth,	18 (62.1)	-
Hindu:	10 (34.5)	-	n (%):		
Number of pregnancies:	2.5 ± 1.7	1-8	-	-	-
Number of live births:	2.4 ± 1.6	1-8	-	-	-
24 h water intake (L):	2.45 ± 1.15	1.25 – 6.5	-	-	-
Arsenic in water (μg/L):	45.4 ± 71.8	5 - 387	-	-	-
SES, Wealth index, n (%):			Food given in past		
1	4 (13.8)	-	24 hours at 6		
2	3 (10.3)	-	months, n (%):		
3	7 (24.1)	-	Breast milk		-
4	4 (13.8)	-	Spoon feeding	26 (89.7)	-
5	4 (13.8)	-	Vitamin	14 (48.3)	-
Missing	7 (24.1)	-	Water	03 (10.3)	-
			Formula milk	38 (96.5)	-
			Fruit juice	14 (48.3)	-
			Semolina	12 (41.4)	-
			Orange coloured food	18 (62.1)	-
			Eggs	04 (13.8)	-
			^*^Sweets	10 (34.5)	-
				11 (37.9)	-

^**§**^Normal water, sugar or glucose water, sugar crystals or misri water, honey, powder milk or pure cow’s milk, infant formula milk; ^*^Chocolate, cake, sweets made of milk product.

Arsenic concentration in human milk was non-normally distributed and skewed to the right at each time point of sample collection. Therefore we performed a non-parametric Kruskal-Wallis test to see whether there was any significant difference in human milk arsenic content according to the household’s drinking water arsenic category (<50 μg/L and ≥50 μg/L); we did not find any significant difference (*P = 0.9, 0.7 and 0.1 at 1, 6 and 9 months, respectively*).

Arsenic concentration in human milk at 1, 6 and 9 months ranged from 0.5 to 8.9 μg/L, 0.5 to 2.32 μg/L and 0.5 to 1.68 μg/L, respectively. The median arsenic concentration in human milk at all three time points remained the same at 0.5 μg/L, 10th-90th percentiles were 0.5 -2.35 μg/L at 1 month, 0.5 – 1.52 μg/L at 6 month and 0.5 – 1.59 μg/L at 9 months (Table 2).

**Table 2:** **Arsenic in breast milk at different time points**

Time point of measurement	Mean ± SD, μg/L	Median, μg/L (range)	10-90th percentile, μg/L
1 month of infants age, (n = 29)	1.12 ± 1.86	0.5 (0.5-8.9)	0.5 – 2.35
6 months of infants age, (n = 25)	0.78 ± 0.45	0.5 (0.5-2.32)	0.5 – 1.52
9 months of infants age, (n = 19)	0.70 ± 0.42	0.5 (0.5-1.68)	0.5 – 1.59

In the univariate analysis, arsenic excretion via human milk did not differ by socio-economic status at any time point except a significant association observed between lowest and middle asset quintile groups at 1 month of child’s age (p < 0.05).

In the mixed model estimates, arsenic concentration in human milk was non significantly reduced between 1 and 6 months and between 6 and 9 months by -0.035 μg/L (95% CI: -0.09 to 0.02). As time progressed, arsenic concentration in children’s urine increased non-significantly by 0.13 μg/L (95% CI: -1.27 to 1.53).

Median arsenic concentration in infant urine at 1 month was 9.2 μg/L and 10th - 90th percentiles were 6 μg/L- 45 μg/L (Table 3). Median concentration of urinary arsenic in infants at 6 months was approximately double the value at 1 month (16.4 μg/L; 10th – 90th percentiles are 6.6 μg/L – 36 μg/L).

**Table 3:** **Arsenic in maternal and infants urine at different time points of measurement**

	Time point of measurement	Mean ± SD, μg/L	Median, μg/L (range)	10-90th percentile, μg/L
Maternal urine	1 month of infants age, (n = 29)	154.8 ± 112.5	134 (5.5-432)	25 -330
	6 months of infants age, (n = 27)	90.2 ± 59.8	72 (10.4-252)	18 -165
Infants urine	1 month of infants age, (n = 29)	18.1 ± 15.1	9.2 (3–54)	6 - 45
	6 months of infants age, (n = 27)	18.8 ± 11.3	16.4 (3–45)	6.6 - 36

**Figure 1:**
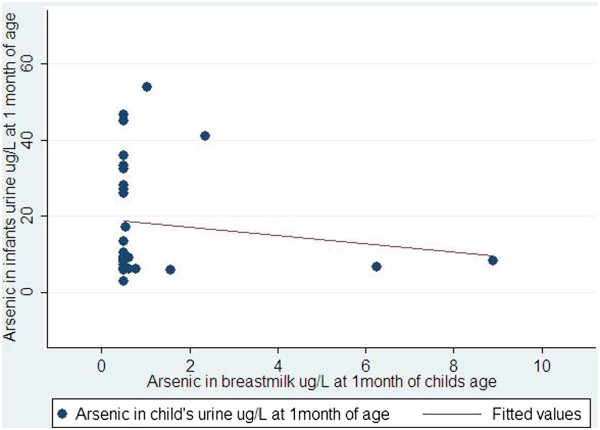
**Correlation between arsenic in breast milk and arsenic in urine of children at 1 month of children’s age.**

**Figure 2:**
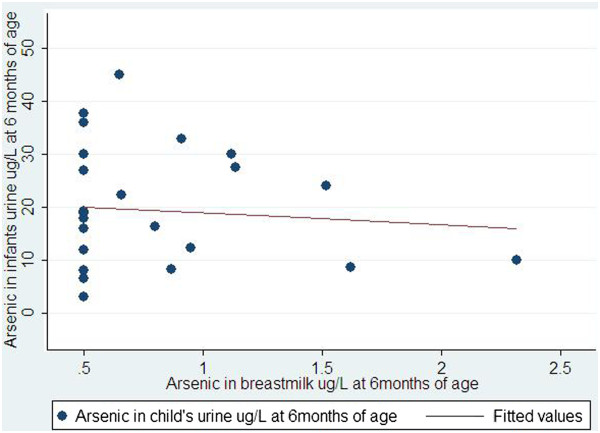
**Correlation between arsenic in breast milk and arsenic in urine of children at 6 month of children’s age.**

Maternal median urinary arsenic concentration at 1 month was 134 μg/L, 10th – 90th percentiles were 25 μg/L – 330 μg/L. At 6 months, median arsenic concentration in maternal urine was 72 μg/L, 10th – 90th percentiles were 18 μg/L – 165 μg/L (Table 3).Maternal urinary arsenic was very weakly correlated with arsenic in human milk at 1 and 6 months of infant’s age (r = 0.13 and 0.21, respectively). Arsenic in human milk at 1 and 6 months was not correlated with that of infant’s urine (r = -0.13 at 1 month and r = -0.09 at 6 month, Figures 1 and 2).

A total of 12 random urine samples at 1 and 6 months of age of infants were analysed for arsenic speciation. Of the infants urine samples at 1 month of age, six out 12 urine samples detected trivalent inorganic arsenic i.e. arsenite (AsIII) above the applied dilution limit of <0.080U (Table 4). The median arsenite concentration in infant’s urine at 1 month of age was 0.71 μg/L (10th – 90th percentiles were 0.09 – 15.4 μg/L). At 6 months, ten of the 12 urine samples from the infants contained arsenite above the detection limit of <0.080U or applied dilution (Table 5). The median urinary arsenite in infants at 6 months of age was 1.6 (10th – 90th percentiles were 0.33 – 3.7 μg/L).

**Table 4:** **Speciation and quantification of arsenic in infant and maternal urine at 1 month of infant’s age**

Arsenic and Arsenic species	Mother			Infant		
	N	Median, in μg/L(10th-90th percentiles)	Range: min – max, in μg/L	N	Median, in μg/L(10th-90th percentiles)	Range: min – max, in μg/L
Total arsenic	11	78 (24–218)	1-224	12	4 (2–29)	1-41
Arsenite (AsIII)	10	4.6 (2.3 – 25.7)	2.06 – 25.9	6	0.71 (0.09 – 15.4)	0.09 -15.4
Arsenate (AsV)	11	2.05 (1.16 – 5.78)	0.32 – 15.7	6	1.37 (0.48 – 3.07)	0.48 – 3.07
Monomethyl Arsonic Acid (MMA)	10	11.64 (2.82 – 47.65)	2.46 – 65.5	10	0.41 (0.10 – 3.97)	0.10 – 4.23
Dimethyl Arsinic Acid (DMA)	11	51 (14.8 – 152)	0.351 – 158	12	3.2 (1.32 – 18.3)	1.29 – 19.8
Arsenobetaine	6	0.76 (0.18 – 5.7)	0.064- 6.21	6	0.20 (0.09 – 0.48)	0.09 – 0.48

**Table 5:** **Speciation and quantification of arsenic in infant and maternal urine at 6 months of child’s age**

Arsenic and Arsenic species	Mother			Infant		
	N	Median, in μg/L(10th-90th percentiles)	Range: min – max, in μg/L	N	Median, in μg/L(10th-90th percentiles)	Range: min – max, in μg/L
Total arsenic	12	50.5 (20 – 87)	14 - 93	12	16.5 (3–27)	2 - 28
Arsenite (AsIII)	12	4.97 (2.74 – 11.6)	0.697 – 24.6	10	1.61 (0.34 – 3.72)	0.33 – 3.8
Arsenate (AsV)	12	1.26 (0.65 – 7.09)	0.517 – 10.7	12	0.40 (0.14 – 1.36)	0.12 – 3.07
Monomethyl Arsonic Acid (MMA)	12	7.41 (2.44 – 11.6)	0.836 – 13.1	12	0.99 (0.31 – 3.01)	0.13 – 3.42
Dimethyl Arsinic Acid (DMA	12	34.65 (14.8 – 63.9)	11.7 – 66.6	12	12.75 (2.1 – 19.7)	1.25 – 20.2
Arsenobetaine	10	0.46 (0.11 – 1.77)	0.096 – 2.21	10	0.24 (0.09 – 5.08)	0.06 – 6.34

Five of the 12 infant urine samples contained pentavalent inorganic arsenic i.e. arsenate (AsV) above the detection limit of <0.33U or applied dilution at 1 month of infant’s age. The median arsenate concentration in infant’s urine at 1 month was 1.37 μg/L (10th – 90th percentiles were 0.48 – 3.07 μg/L). On the other hand, all 12 infantile urine samples contained arsenate above the detection limit of <0.33U or applied dilution at 6 months. Median urinary arsenate concentration at 6 months was 0.40 (10th – 90th percentiles were 0.14 – 1.36 μg/L).

As shown in Tables 4 and 5, other urinary arsenic metabolites present in infant and maternal urine are monomethyl arsonic acid (MMA), dimethyl arsinic acid (DMA) and arsenobetaine (AsB). DMA constitutes the major arsenic species in urine both at 1 and 6 months. Excretion of DMA and AsB in infant urine is increased at 6 months compared to 1 month while it is decreased in maternal urine.

Since we collected drinking water samples from households at initial contact when we advised them not to use water from a tubewell that were marked with red colour and, urine samples at 1 and 6 months of infant’s age therefore, we were interested to see whether mean arsenic concentration in maternal urine differed by household drinking water arsenic categories. Mean total maternal urinary arsenic concentration was lower at both time points in the mothers whose household drinking water contained high arsenic (≥50 μg/L) compared to the household’s drinking water of the mothers that contain low (<50 μg/L) arsenic. Mean of urinary arsenic concentrations at 1 month of infant’s age were 168.1 μg/L and 103.6 μg/L in the mothers whose household drinking water contain <50 and ≥50 μg/L of arsenic while mean of the urinary arsenic concentrations at 6 month of infant’s age were 92.7 μg/L and 81.3 μg/L in the mothers whose household drinking water contain <50 and ≥50 μg/L of arsenic. Considering the low maternal urinary arsenic content in the households with ≥50 μg/L of arsenic in their drinking water, we modelled simple linear regression to see whether the decline was significant between hosuehold’s drinking water arsenic categories. As expected, we did not find any difference since the households might have changed their source of drinking water from the point of household’s drinking water sample collection to urine sampling.

## Discussion

This study primarily demonstrates a low median arsenic content in breast milk at 1, 6 and 9 months, despite high arsenic exposure via drinking water. About 69%, 56% and 74% of breast milk samples contained arsenic concentration at the level of 0.5 μg/L at 1, 6 and 9 months of infant’s age, respectively, while about 80% of the breast milk samples contained arsenic ≤1 μg/L at each time point. Maternal and infant urine contained high amount of arsenic irrespective of their drinking water arsenic exposure categories. Inorganic and methylated arsenic are the major constituents of total urinary arsenic in both mother and infant and dimethyl arsinic acid is the predominant species. Arsenic excretion in breast milk did not show any association with parents’ education, occupation and the household’s socio-economic status. There was no correlation between arsenic in breast milk and maternal or infant urine at any time point.

Information on low excretion of arsenic in breast milk has been reported previously, although there is still a scarcity of adequate data. Maternal exposure to high arsenic did not translate into passing significant amount of inorganic arsenic in breast milk [23]. One study reported that breast milk in the first 3 months of lactation contained arsenic within the normal safety limit [24]. Other studies claimed a protective effect of breastfeeding as arsenic excretion via breast milk was very low or below the level of quantification and mostly of the trivalent form of inorganic arsenic [3, 7, 25]. Also, a recent Taiwanese study demonstrated similar findings and recommended encouragement of breastfeeding [26]. Our study concurs that arsenic content in breast milk at each time point of collection (1, 6 and 9 months) is low and declines over time.

Contrary to the low arsenic concentration in breast milk, urinary arsenic in these infants at exclusively breastfeeding age (0–6 month) was relatively high. Urinary arsenic concentrations depend on many other exogenous factors, such as arsenic content in drinking water, amount of water consumption, arsenic exposure from multiple sources and duration between exposure to arsenic and collection of urine samples as 60-75% of ingested arsenic is excreted in 3 days via urine [27, 28]. We observed very high DMA constituents in infant’s urine compared to MMA at 1 and 6 months of child’s age (Tables 2 and 3) and these findings are quite similar to another study conducted in Bangladesh [7]. Concha et al. also reported similar findings of high DMA and low MMA in urine of new born babies in the Andes, Argentina and this was consequent to transfer of DMA to foetus by the mother as a result of an efficient arsenic methylation at later stage of pregnancy [15]. In response to the foetal demand of choline for the development of their brain, new synthesis of choline by phosphatidyletanolamine methyltransferase occurred, subsequently causing an efficient arsenic methylation in later pregnancy [29]. In the case of low folate intake, folate-linked remethylation of homocystine for its removal from body does not occur and removal of homocystine is a prerequisite for an efficient one carbon metabolism of arsenic methylation, and endogenous production of choline is the only alternate for arsenic methylation in folate deficient people [30]. In this study we did not collect data on maternal or foetal folate intake although it was likely that the study population was deficient in folate [31]; therefore the alternate pathway i.e. endogenous production of choline, might be the predominant route of arsenic methylation in the study population and subsequently an increased methylated arsenic content was measured in both maternal and children urine. Higher urinary arsenic in children indicates additional means of arsenic exposure other than breastfeeding and the mechanism of arsenic excretion in breast milk might be different from excretion via urine [17]. High excretion of methylated arsenic via urine in children is induced by high amount of choline and folate content in mature breast milk in the first few postpartum months [32, 33].

We surmise that the relatively high urinary arsenic in infants is related to the intake of food and drink other than breast milk. We are doing further work involving deuterium oxide (D_2_O) dose to mother studies [20] to explore this hypothesis. Besides, it is not possible to completely rule out the chances of urine sample contamination with arsenic polluted water that are used for bathing and cleaning the children, although the effect of such contamination is likely to be very smaller; for example, if 10 ml of urine is contaminated with 0.1 ml of water with 500 μg As/L concentration, there is a chance of increasing the concentrations by 5 μg/L only [7].

In our study, water arsenic concentration was used to characterise arsenic exposure, although the level of exposure might have changed in the households following our advice on not using any tubewell water that were marked with ‘red colour’ (tubewell marked with red colour when this contains ≥50 μg of arsenic per litre of water) at the time of initial recruitment of participants. Therefore the water arsenic exposure category for ≥50 μg/L might not reflect the actual consumption over the successive period of the study thus in the biological sample analysis. As expected, we did not find any difference in maternal urinary arsenic levels by arsenic exposure categories in this study. Urinary arsenic levels are good indicators for recent exposure therefore, differences in maternal urinary arsenic level by arsenic exposure categories are not likely to be the effect of changing drinking water sources. However, we do not have data on how many households changed their source of drinking water during the study period and the urinary arsenic levels presented here are unadjusted by creatinine thus implying potential limitation. Further limitation includes smaller sample size and not using other samples such as nail, hair, blood or skin for arsenic detection. In contrary, urinary arsenic speciation and collection of infants urine using PUC are the strengths of this study.

In this study, we observed no correlation between arsenic in breast milk and infant urine. Concha G et al., observed a similar findings [23]. We found a low concentration of arsenic passing in breast milk even in those who passed high arsenic in their urine, although arsenic transport mechanism through breast milk is not fully understood. Breast milk contains low arsenic concentration and this may be due to an efficient maternal methylation of inorganic arsenic [7]. Arsenic in trivalent form (AsIII), mainly passes through breast milk and possibly is the only arsenic metabolite that is protonated at physiologic pH (pK_a1_ of arsenous acid = 9.2), via a transporter called aquaglyceroporins, which transports AsIII in most organisms [7, 34, 35] and found in rat mammary gland during lactation [36]. One study indicated that arsenic excretion in breast milk varied greatly by individual and therefore breast milk values did not cluster with values from water or other biological samples [17]. Likewise, despite high exposure, low arsenic concentration in breast milk indicates that mammary gland acts as an effective filter that limits excretion of arsenic via breast milk. The concentrations seems to be much lower than World Health Organization’s maximum permissible limit (WHO Permissible Limit 15 μg/kg-bw/week) [37].

## Conclusions

Arsenic in breast milk was very low in women living in arsenic contaminated areas. Breast milk arsenic did not correlate with infants’ urinary arsenic, likely because of supplemental feeding from other sources. Our data support the recommendation that breastfeeding is safe for infants living in arsenic contaminated areas.
